# Natural *SEL1L* variants rescue a model of NGLY1 deficiency and modify ERAD function and proteasome sensitivity

**DOI:** 10.1371/journal.pgen.1011823

**Published:** 2025-08-07

**Authors:** Travis K. Tu’ifua, Clement Y. Chow

**Affiliations:** Department of Human Genetics, University of Utah School of Medicine, Salt Lake City, Utah, United States of America; HudsonAlpha Institute for Biotechnology, UNITED STATES OF AMERICA

## Abstract

N-glycanase 1 (NGLY1) deficiency is an ultra-rare disease caused by autosomal recessive loss-of-function mutations in the *NGLY1* gene. NGLY1 removes N-linked glycans from glycoproteins in the cytoplasm and is thought to help clear misfolded proteins from the endoplasmic reticulum (ER) through the ER associated degradation (ERAD) pathway. Despite this, the physiological significance of NGLY1 in ERAD is not understood. The best characterized substrate of NGLY1 is NRF1, a transcription factor that upregulates proteasome expression and the proteasome bounce-back response. We previously performed a genetic modifier screen using a *Drosophila* model of NGLY1 deficiency and identified potential modifiers that alter the lethality of the model. We identified two protein-coding variants in *Hrd3*/*SEL1L*: *S780P* and *Δ806–809*. Both variants are localized to the SEL1L cytoplasmic tail, an uncharacterized domain. SEL1L is a component of the ERAD complex that retrotranslocates misfolded proteins from the ER to the cytoplasm for degradation. We used CRISPR to generate fly lines carrying these *SEL1L* variants in a common genetic background and tested them with our model of NGLY1 deficiency. Validating our previous screen, the *SEL1L*^*S780P*^ and *SEL1L*^*Δ806-809*^ variants increased the survival of the NGLY1 deficiency model, compared to the *SEL1L*^*S780*^ variant. To determine how these *SEL1L* variants were modifying lethality in NGLY1 deficiency, we interrogated the ERAD and NRF1 signaling pathways. We found that the *SEL1L*^*S780P*^ and *SEL1L*^*Δ806-809*^ variants improve resistance to ER stress, with enhanced ERAD function as a likely contributing mechanism. This effect depends on NGLY1 activity, further implicating NGLY1 in general ERAD function. We also found that, in heterozygous *NGLY1* null flies, these variants protect against some defects like increased lethality caused by proteasome inhibition. These results provide new insights into the role of SEL1L in the disease pathogenesis of NGLY1 deficiency. *SEL1L* is a strong candidate modifier gene in patients, where variability in presentation is common.

## Author’s summary

NGLY1 deficiency is a debilitating rare genetic disorder. There are currently no treatment options for NGLY1 deficiency and NGLY1 biology remains poorly understood. We previously performed a genetic modifier screen in a *Drosophila* model of NGLY1 deficiency and identified a number of candidate modifier genes that impacted the survival of our model. Modifier genes can help reveal NGLY1 biology and NGLY1 deficiency disease pathogenesis. In this study, we follow-up on two natural protein-coding variants of *Hrd3* (the fly version of the human gene, *SEL1L*) that increased the survival to adulthood of our NGLY1 deficiency model. SEL1L is a critical component of an important quality control pathway called the endoplasmic reticulum associated degradation (ERAD) pathway. We discovered that these *SEL1L* variants increased resistance to ER stress and may enhance ERAD function. These *SEL1L* variants also modify NGLY1 deficiency sensitivity to proteasome inhibition. This study confirms *SEL1L* as an important modifier gene of NGLY1 deficiency. Further study of the ERAD and proteasome degradation pathways may reveal additional candidate modifier genes of NGLY1 deficiency and potential targets for therapeutic development.

## Introduction

N-glycanase 1 (NGLY1) deficiency is an ultra-rare disease and the first identified congenital disorder of deglycosylation (CDDG). The disease is caused by autosomal recessive loss-of-function mutations in the *NGLY1* gene [1,2]. NGLY1 is a cytosolic deglycosylating enzyme that removes N-linked glycans from proteins. NGLY1 is thought to be a component of the endoplasmic reticulum (ER) associated degradation (ERAD) pathway, an important cellular quality control mechanism which removes misfolded proteins from the ER to the cytosol for degradation [3,4]. However, the loss of NGLY1 shows little effect on ERAD and does not prevent the degradation of misfolded proteins [5–7]. Therefore, despite its known function as a deglycosylating enzyme, the physiological significance of NGLY1 and disease pathogenesis remains poorly understood.

NGLY1 deficiency is marked by extensive phenotypic heterogeneity, even among patients with identical *NGLY1* mutations [2,8], suggesting the presence of genetic modifiers. In a previous genetic screen, we identified 61 potential modifier genes that were associated with changes in survival in our *Drosophila* model of NGLY1 deficiency [9]. From this screen, our top hit was *Ncc69* (*Drosophila* ortholog for human *NKCC1/2*), which encodes for a conserved ion transporter and we showed that it is both a substrate of NGLY1 and a modifier of NGLY1 deficiency [9]. Another interesting candidate modifier gene we identified in the screen was *Hrd3* (hereon referred to by the human ortholog *SEL1L*). Through a genome-wide association study (GWAS), we identified a natural missense variant in *SEL1L* that was associated with increased survival in the *Drosophila* NGLY1 deficiency model [9]. This *SEL1L* variant is a substitution of serine 780 for a proline (*SEL1L*^*S780P*^). Additionally, we identified a private protein-coding deletion in the strain showing near complete rescue of NGLY1 deficiency lethality. This second variant is a deletion of amino acids 806–809 (*SEL1L*^*Δ806-809*^). Both variants are 26 amino acids apart and are located in the cytoplasmic tail of SEL1L.

SEL1L is a single-pass ER membrane protein and a critical, well-established component of ERAD. ERAD functions alongside other quality control mechanisms such as the unfolded protein response (UPR) and autophagy to maintain ER homeostasis and prevent ER stress [10–12]. The SEL1L-Hrd1 ERAD complex is the most conserved branch of ERAD from yeast to humans and translocates misfolded proteins from the ER to the cytosol for proteasomal degradation [13–15]. The luminal domain of SEL1L assists in the recognition of ERAD substrates in the ER lumen and the transmembrane domain helps move proteins through the ER membrane to the cytosol [16]. The cytoplasmic tail of SEL1L is a highly disordered region across species and its function is unknown.

In previous studies, both *SEL1L* and *NGLY1* were identified as genetic modifiers of NRF1, a transcription factor responsible for the proteasome bounce-back response [17,18]. NRF1 is co-translated and glycosylated in the ER before being retrotranslocated to the cytosol, a process that requires ERAD machinery, including SEL1L, HRD1, and p97 [13,17,19]. Once in the cytosol, NRF1 is deglycosylated by NGLY1, which edits asparagine residues to aspartate and is required for NRF1 activation, localization, and transcriptional function [18,20]. The protease DDI1/2 also cleaves NRF1 to promote its activation and nuclear translocation [20]. However, some studies suggest that full-length NRF1 can reach the nucleus in the absence of DDI1/2 activity, indicating that its role may be context or species-specific [17,18]. Although SEL1L has been genetically linked to NRF1 activity and is a critical component of the Hrd1 ERAD complex, its specific mechanistic role in NRF1 retrotranslocation has not been fully delineated [13,17,21]. Nevertheless, SEL1L is strongly implicated in mediating NRF1 dislocation from the ER and may modulate the efficiency of NRF1 activation under proteasome stress conditions. Under healthy, homeostatic conditions, activated NRF1 is constitutively degraded by the proteasome. Under conditions of proteasomal stress, however, NRF1 is not degraded, accumulates in the cytosol, and is transported to the nucleus where it acts as a transcription factor and upregulates genes that increase proteasome function, including proteasome subunit genes [17,18,22]. This activation of NRF1 is known as the proteasome bounce-back response. NGLY1 and ERAD machinery are necessary for NRF1 activation and the loss of either prevents the proteasome bounce-back response [17,18].

In this study, we characterized the functional consequences of the two new *SEL1L* variants identified in our NGLY1 deficiency genetic screen. We placed the *SEL1L* variants on an isogenic background to test the effects each variant has on both SEL1L and NGLY1. The *SEL1L* variants increased eclosion rates and survival to adulthood in our NGLY1 deficiency model, validating the observations from the modifier screen. The *SEL1L* variants also enhance ERAD in an NGLY1-dependent manner and provide a protective fitness advantage during proteasome inhibition. Our results suggest that *SEL1L* is a modifier of *NGLY1* and that interactions between *SEL1L*, *NGLY1*, and *NRF1* may underlie these observed changes in fitness. These genetic interactions are potential targets for NGLY1 deficiency treatment.

## Results

### *SEL1L*^*S780P*^ and *SEL1L*^*Δ806-809*^ variants increase eclosion rates and survival to adulthood of NGLY1 deficiency model

**Fig 1:**
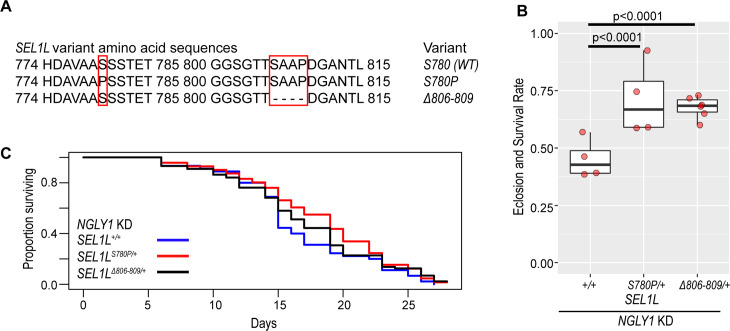
*SEL1L* variants increase NGLY1 deficiency eclosion rates and survival to adulthood. **(A)** Amino acid sequences for the three *SEL1L* variant alleles. Red boxes highlight differences between alleles. **(B)** *SEL1L*^*(S/P, +/+)*^ and *SEL1L*^*Δ806-809/+*^ genotypes significantly increase eclosion rates and survival to adulthood (~70%) with *NGLY1* knockdown when compared to *SEL1L*^*+/+*^ (45%, p < 0.0001). Chi-squared test. **(C)** Lifespan of *NGLY1* KD flies shows no significant difference in survival to adulthood between *SEL1L* genotypes. Cox proportional hazard regression analysis.

In a previous study, we crossed our NGLY1 deficiency *Drosophila* model, which uses the *GAL4/UAS* system to ubiquitously express RNAi against *NGLY1*, with nearly 200 strains of the *Drosophila* Genetic Reference Panel (DGRP) [9,23]. On a standard laboratory background, the NGLY1 deficiency model has ~ 30% survival to adulthood [7,9]. In the DGRP strains, eclosion rates and survival to adulthood of the NGLY1 deficiency model ranged from 0 to 100%, indicating that lethality is highly modifiable by genetic background. We performed a GWAS to identify candidate modifier genes associated with increased survival to adulthood. One of the top associated variants was the S780P missense variant in *SEL1L*. The *SEL1L*^*S780P*^ minor allele was associated with increased eclosion rates and survival to adulthood of the NGLY1 deficiency model, compared to the common *SEL1L*^*S780*^ allele. We also discovered that the DGRP strain (DGRP strain 379) with a nearly 100% eclosion rate and survival to adulthood in the screen harbored a private *SEL1L* variant, resulting in the deletion of amino acids 806–809 (*SEL1L*^*Δ806-809*^). This strain also carries the more common *S780* allele. *SEL1L*^*Δ806-809*^ was not formally identified through the GWAS because it is a private variant in a single DGRP strain. Because these two variants were both in the cytoplasmic tail of SEL1L, a functionally uncharacterized region of the protein, we sought to understand how these variants were affecting eclosion rates and survival to adulthood of the NGLY1 deficiency model. We used CRISPR to place each of the *SEL1L* variants (Fig 1A) onto the same isogenetic background, creating three strains that are homozygous for *SEL1L*^*S780*^, *SEL1L*^*S780P*^*,* or *SEL1L*^*Δ806-809*^. This allowed us to test for phenotypic differences specific to each *SEL1L* variant.

In our original screen, we crossed a strain carrying both a GAL4 and *NGLY1 RNAi* transgene with strains of the DGRP. Lethality was scored based on eclosion rates and survival to adulthood of the F1 flies, which had half of their genomes from the *NGLY1* RNAi strain and half from the different DGRP strains. Because the *NGLY1* RNAi strain is homozygous for the common, wildtype *SEL1L*^*S780*^ allele, any new *SEL1L* variant introduced by the DGRP strain in the F1 generation was heterozygous with the *SEL1L*^*S780*^ allele. For clarity, we will refer to the more common major S780 allele as “wildtype” or *SEL1L*^*+/+*^. Therefore, the relevant *SEL1L* genotypes from our screen are: *SEL1L*^*+/+*^, *SEL1L*^*S780P/+*^, or *SEL1L* ^*Δ806-809/+*^. We focus our analyses on these three *SEL1L* genotypes throughout this study.

Based on the original DGRP screen, we expected that the *SEL1L*^*S780P/+*^ and *SEL1L*^*Δ806-809/+*^ genotypes would increase eclosion rates and survival to adulthood of the NGLY1 model, compared to the wildtype *SEL1L* genotype. To validate the results of the screen, we crossed the same *NGLY1* RNAi strain used in the modifier screen with each of our new *SEL1L* variant CRISPR strains to generate flies that have the exact *SEL1L* genotypes from the screen. The eclosion rates and survival to adulthood was determined in the same manner as the original screen, by dividing the number of *NGLY1* knockdown flies by the largest balancer class in its cross [9]. There were significantly increased eclosion rates and survival to adulthood of *NGLY1* knockdown flies with the *SEL1L*^*S780P/+*^ and *SEL1L*^*Δ806-809/+*^ genotypes compared to the *SEL1L*^*+/+*^ genotype (Fig 1B and S1 Data). The *SEL1L*^*+/+*^ genotype had a 45% eclosion rate and survival to adulthood compared to ~70% in the *SEL1L*^*S780P/+*^ (p < 0.001) and *SEL1L*^*Δ806-809/+*^ (p < 0.001) genotypes. This result nicely replicates our previous genetic screen that showed increased eclosion rates and survival to adulthood in the DGRP lines with these particular *SEL1L* variants and suggests that the *SEL1L*^*S780P*^ and *SEL1L*^*Δ806-809*^ alleles are protective against NGLY1 deficiency. We also evaluated the lifespan of these surviving *NGLY1* knockdown flies, but found no significant differences between the *SEL1L*^*S780P/+*^ and *SEL1L*^*Δ806-809/+*^ and the *SEL1L*^*+/+*^ genotype (Fig 1C and S1 Data), suggesting that the interaction occurs during development.

### *SEL1L* variants impact sensitivity to proteasome inhibition in *NGLY1*^*+/-*^ flies

We next interrogated pathways that involve both SEL1L and NGLY1 to understand how these *SEL1L* variants are protecting against NGLY1 deficiency. Both *NGLY1* and *SEL1L* were previously identified as modifier genes of *NRF1*, which encodes for an important transcription factor that upregulates proteasome genes in response to proteasomal stress [17,18]. The importance of NGLY1 in NRF1 function is well established. NGLY1 mutants have reduced proteasome function and are exquisitely sensitive to proteasome stress because NRF1 is not processed [17,18,24,25]. Previous studies have shown that heterozygous *NGLY1* null larvae, which are otherwise normal, are sensitive to proteasome inhibition, leading to larval size defects [24,25]. Although *SEL1L* was identified as a genetic modifier of NRF1, its role in NRF1 signaling has not been determined. We hypothesized that these *SEL1L* variants would affect NRF1 signaling and modify phenotypes in an NGLY1 deficiency model.

We tested whether the *SEL1L* variants affect proteasome sensitivity in NGLY deficient *Drosophila* using the proteasome inhibitor bortezomib (BTZ). Homozygous *NGLY1* null *Drosophila* are embryonic lethal, but heterozygous *NGLY1* null flies are phenotypically normal when unchallenged. We used heterozygous *NGLY1* null flies as a model of NGLY1 deficiency because of their known increased sensitivity to proteasome inhibition [24,25]. Because the *NGLY1* null strain also carries the common wildtype S780 allele, when we cross this strain with our CRISPR generated *SEL1L* variant strains, we generate heterozygous *NGLY1* null flies with the same *SEL1L* genotypes to what we tested in the *NGLY1* knockdown model: *SEL1L*^*+/+*^, *SEL1L*^*S780P/+*^, or *SEL1L*^*Δ806-809/+*^.

**Fig 2:**
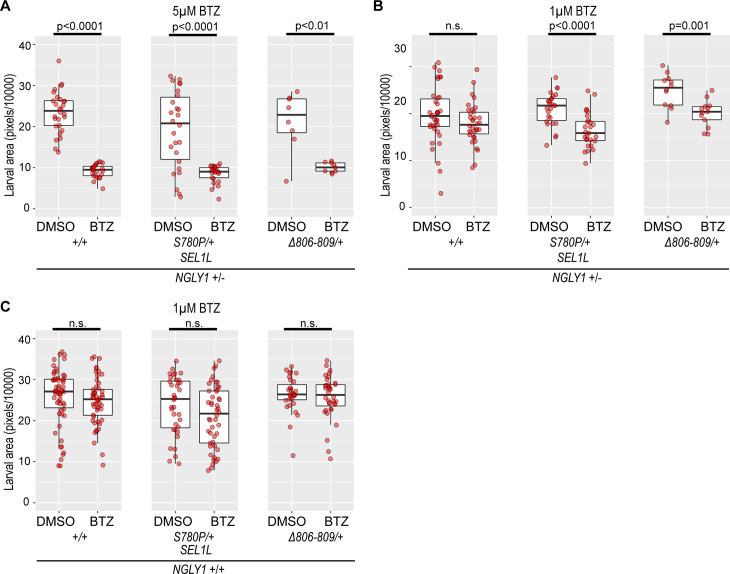
*SEL1L* variants affect proteasome inhibition sensitivity. **(A)** *NGLY1 + /-* larvae are smaller when treated with 5μM BTZ, but there is no effect of *SEL1L* genotype (Larval size on DMSO: *SEL1L*^*+/+*^ 23.59 ± 4.97, *SEL1L*^*S780P/+*^ 19.37 ± 9.16, and *SEL1L*^*Δ806-809/+*^ 21.32 ± 6.73. Decreased larval size on BTZ: *SEL1L*^*+/+*^ 9.19 ± 1.61, p < 0.0001; *SEL1L*^*S780P/+*^ 8.55 ± 2.04, p < 0.0001; and *SEL1L*^*Δ806-809/+*^ 10.07 ± 1.14, p < 0.01). **(B)** When treated with 1μM BTZ, the *NGLY1 + /-* larvae show a *SEL1L* genotype-dependent decrease in size. While *SEL1L*^*+/+*^ showed no change between DMSO (19.65 ± 5.98) and BTZ (17.88 ± 4.32), *SEL1L*^*S780P/+*^ (DMSO 21.03 ± 3.42, BTZ 16.45 ± 3.65, p < 0.0001) and *SEL1L*^*Δ806-809/+*^ (DMSO 24.92 ± 3.39, BTZ 20.08 ± 2.69, p = 0.001) genotypes were smaller when treated with BTZ compared to DMSO treated larvae. **(C)** *NGLY1 WT* larvae show no significant decrease in size with 1μM BTZ treatment, regardless of *SEL1L* genotype (Larval size on DMSO: *SEL1L*^*+/+*^ 25.75 ± 6.91, *SEL1L*^*S780P/+*^ 23.74 ± 6.94, and *SEL1L*^*Δ806-809/+*^ 26.42 ± 4.29; larval size on BTZ: *SEL1L*^*+/+*^ 24.75 ± 5.63, *SEL1L*^*S780P/+*^ 20.96 ± 7.38, and *SEL1L*^*Δ806-809/+*^ 25.70 ± 5.23).

Heterozygous *NGLY1* null *Drosophila* larvae develop smaller when exposed to proteasome inhibition, compared to *NGLY1* wildtype and DMSO-treated heterozygous *NGLY1* null controls [24,25]. In previous studies, heterozygous *NGLY1* null larvae, when exposed to 5μM BTZ, are significantly smaller than DMSO-treated heterozygous *NGLY1* null larvae [25]. We observed an equally strong decrease in larval size with the treatment of 5μM bortezomib in all heterozygous *NGLY1* null larvae compared to DMSO controls, regardless of *SEL1L* genotype (Fig 2A and S2 Data). *SEL1L* genotype does not impact the size defects induced by 5μM BTZ in *NGLY1* heterozygous null larvae.

To determine whether there might be more subtle effects, we treated the heterozygous *NGLY1* null larvae with a lower concentration of 1μM BTZ. The *SEL1L*^*+/+*^ larvae showed no significant size differences between BTZ and DMSO treatments. However, *SEL1L*^*S780P/+*^ (p < 0.0001) and *SEL1L*^*Δ806-809/+*^ (p = 0.001) larvae had significant decreases in larval size with 1μM BTZ treatment compared to DMSO (Fig 2B and S2 Data). This indicates that larval size in heterozygous *NGLY1* null larvae carrying the *SEL1L*^*S780P/+*^ and *SEL1L*^*Δ806-809/+*^ genotypes are particularly sensitive to proteasome inhibition compared to *SEL1L*^*+/+*^. On an NGLY1 wildtype background, the different *SEL1L* genotypes showed no larval size changes with the 1μM BTZ treatment (Fig 2C and S2 Data).

### *SEL1L* variants increase survival of *NGLY1*^*+/-*^ flies in response to proteasome inhibition

**Fig 3:**
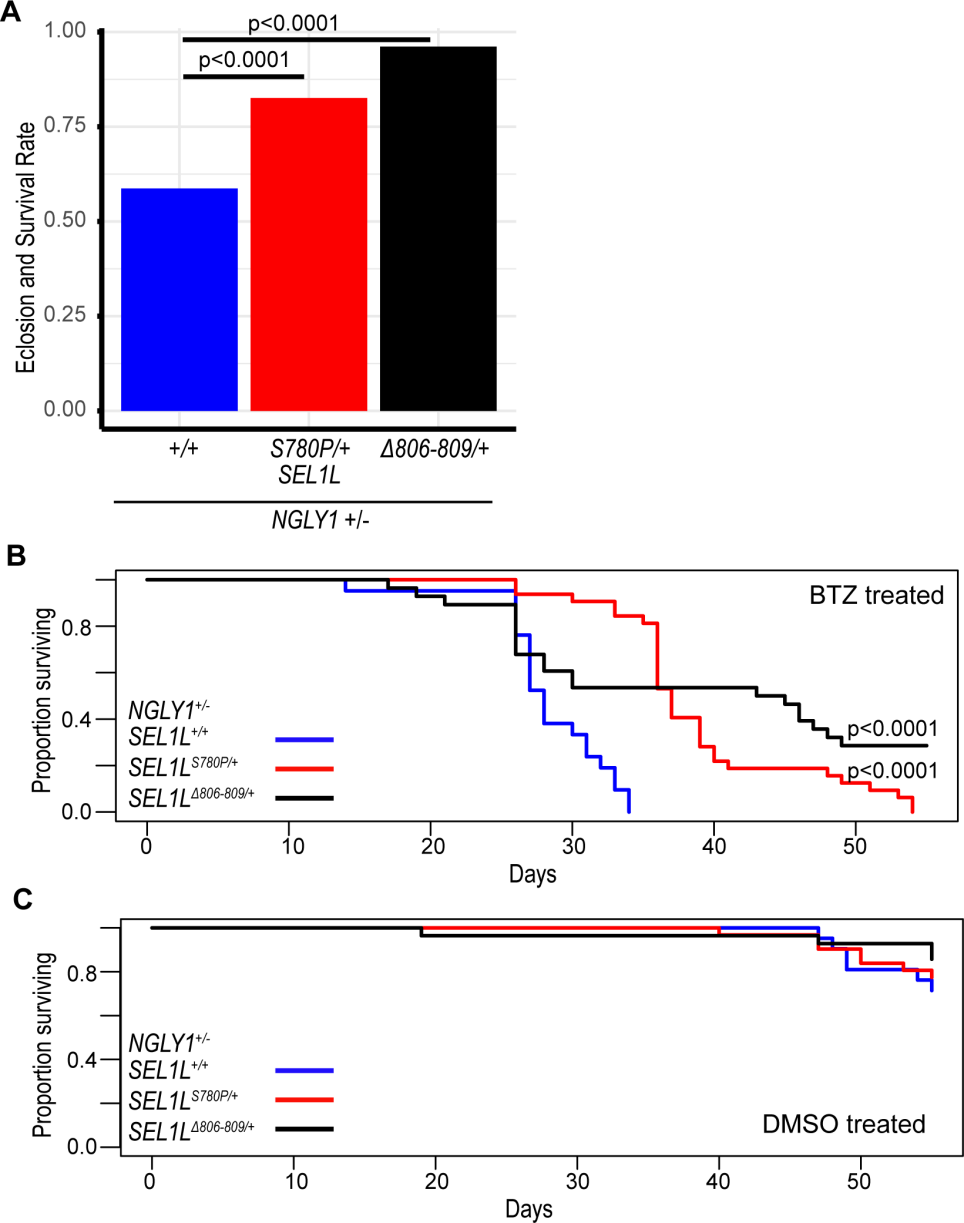
*SEL1L* variants increase survival under proteasome inhibition. **(A)** When treated with 1μM BTZ, *NGLY1 + /-* larvae eclose at significantly higher rates when carrying the *SEL1L*^*S780P/+*^ (83%, p < 0.0001) and *SEL1L*^*Δ806-809/+*^ (96%, p < 0.0001) genotypes compared to the *SEL1L*^*+/+*^ genotype (59%). **(B)** When treated with 1μM BTZ, adult *NGLY1 + /-* flies with the *SEL1L*^*S780P/+*^ (p < 0.0001) and *SEL1L*^*Δ806-809/+*^ (p < 0.0001) genotypes live longer than the *SEL1L*^*+/+*^. Cox proportional hazard regression analysis. **(C)** There is no significant difference in survival between adult *NGLY1 + /-* flies treated with DMSO regardless of *SEL1L* genotype.

To further examine the impact of the *SEL1L* variants on NRF1 signaling, we tested other phenotypes affected by NGLY1 deficiency and proteasome inhibition. When heterozygous *NGLY1* null larvae were treated with 1μM BTZ, we observed a *SEL1L* variant specific effect on survival through eclosion to adulthood. The *SEL1L*^*S780P/+*^ and the *SEL1L*^*Δ806-809/+*^ larvae showed higher eclosion rates and survival to adulthood at 83% (p < 0.0001) and 96% (p < 0.0001), respectively, compared to the *SEL1L*^*+/+*^ flies at 59% (Fig 3A and S3 Data). *SEL1L*^*+/+*^ larvae treated with bortezomib showed high rates of non-eclosed and partially eclosed flies. The improved eclosion rates of the *SEL1L*^*S780P/+*^ and the *SEL1L*^*Δ806-809/+*^ genotypes suggests a protective effect of the *SEL1L*^*S780P*^ and *SEL1L*^*Δ806-809*^ variants against NGLY1 deficiency and proteasome inhibition during larval development. Importantly, when *NGLY1* wildtype larvae were raised on 1μM BTZ, we observed no lethality and nearly 100% eclosion of flies, regardless of *SEL1L* genotype, demonstrating that lethality to BTZ is NGLY1-dependent (S1 Fig and S3 Data). *SEL1L*^*S780P/+*^ and *SEL1L*^*Δ806-809/+*^ genotypes provide near complete rescue of the heterozygous *NGLY1* null larvae lethality induced by 1μM BTZ.

To understand if the *SEL1L* variants impact NRF1 signaling in adult flies, we treated heterozygous *NGLY1* null adult flies with bortezomib or DMSO and observed their long-term, adult survival. The adult flies were raised on DMSO during their larval stages and were either maintained on DMSO or BTZ food. *SEL1L*^*+/+*^ adult flies showed significantly decreased survival time on BTZ compared to the *SEL1L*^*S780P/+*^ (p < 0.0001) and the *SEL1L*^*Δ806-809/+*^ (p < 0.0001) genotypes (Fig 3B and S3 Data). The increased survival times of the bortezomib-treated *SEL1L*^*S780P/+*^ and *SEL1L*^*Δ806-809/+*^ genotypes indicate that these variants are protective against proteasome inhibition during adulthood. When treated with DMSO, heterozygous *NGLY1* null adult flies displayed no significant differences in long-term survival, regardless of *SEL1L* variant genotypes (Fig 3C and S3 Data). These experiments demonstrate that the sensitivity to proteasome inhibition in NGLY1 deficiency is modified by these *SEL1L* variants, both during larval development and in adulthood. The effects of these *SEL1L* variants on proteasome sensitivity are only observed when NGLY1 function is reduced, indicating that the interaction between SEL1L and proteasome stress responses is contingent on the loss of NGLY1 activity.

### *SEL1L* variants do not impact the expression of NRF1 target genes

In response to proteasome inhibition, NRF1 upregulates proteasome genes [22]. To test for changes in NRF1 signaling in our *SEL1L* variant flies, we examined the expression of several proteasomal subunit genes: *prosalpha6*, *prosalpha3*, *prosbeta2*, *prosbeta4*, and *prosbeta5*. These five genes were among other proteasome genes that we previously identified as downregulated in our NGLY1 deficiency fly model [7]. We treated flies with BTZ to induce NRF1 signaling. When we compared expression of the proteasome genes, in whole flies, between *NGLY1* wildtype and heterozygous *NGLY1* null adults, we mostly observed no differences in gene expression across each condition and *SEL1L* genotype (S2A Fig and S4 Data). As expected, we observed increases in proteasome gene expression with the treatment of BTZ compared to DMSO; however, we found no *SEL1L* variant specific differences in gene expression in either the *NGLY1* wildtype or heterozygous *NGLY1* null flies (S2B Fig and S4 Data). While this is unexpected in light of the previous phenotypic data we present, it is possible that there are specific effects in different tissues that are missed when we examine expression in whole flies. To date, it is unknown which tissues contribute to the lethality in NGLY1 deficiency flies and more work is needed to determine which tissues are most impacted by proteasome inhibition.

### *SEL1L*^*S780P*^ and *SEL1L*^*Δ806-809*^ variants enhance ERAD in an ER stress model

SEL1L is an integral component of the Hrd1 ERAD complex, which retrotranslocates misfolded proteins from the ER lumen and ubiquitinates them for degradation by the proteasome [10,13,15]. Although its role in ERAD remains unclear, NGLY1 has been shown to physically interact with proteins in the ERAD complex, including Derlin-1 and VCP [26,27]. The deglycosylation of ERAD substrates by NGLY1 is thought to prepare misfolded glycoproteins for proteasomal degradation [5,28,29].

We crossed a *Drosophila* eye model of ER stress with the *SEL1L* strains to determine the variant-specific effects on ERAD. In this model, a transgene carries an eye-specific GAL4 (*GMR-GAL4*) that drives the overexpression of a mutant misfolded rhodopsin protein (encoded by *UAS-Rh1*^*G69D*^) that constitutively misfolds and induces degeneration in the developing larval eye disc [30–33]. The misfolded rhodopsin protein leads to chronic ER stress in the eye disc, cell death, and a rough eye phenotype in adult flies. These eyes are also significantly smaller than wildtype eyes. This model is sensitive to changes in ERAD function and increased ERAD function is protective against eye degeneration [30]. In larval eye discs, overexpressing Hrd1, the ERAD protein essential for transporting misfolded proteins out of the ER, nearly completely restored the normal appearance of the eye [30]. Increasing Hrd1 levels enhances ERAD, helping to clear misfolded rhodopsin, which in turn prevents ER stress and protects against degeneration. Conversely, knockdown of ERAD components in this model leads to increased eye degeneration [30]. Because SEL1L is a component of the Hrd1 ERAD complex, modifying SEL1L should similarly affect phenotypes in the ER stress eye model.

**Fig 4:**
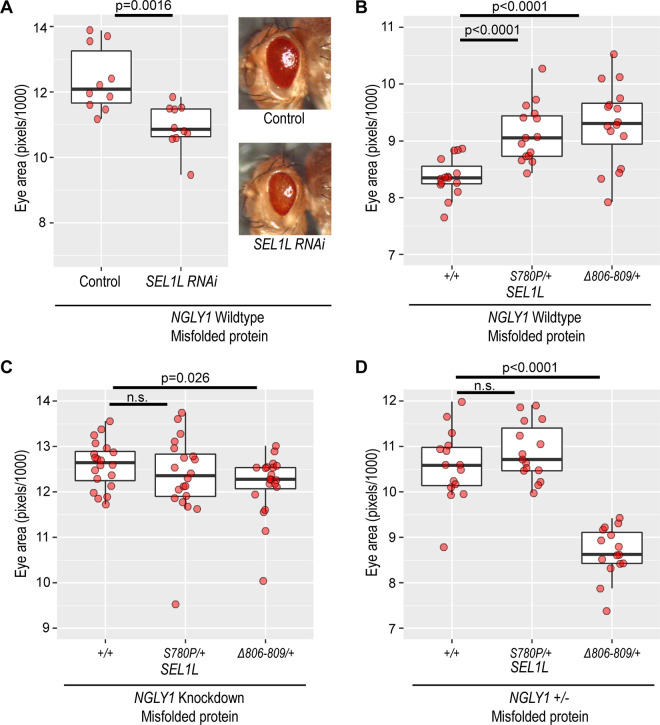
SEL1L variants enhance resistance to ER stress in an NGLY1-dependent manner. To induce ER stress and test ERAD, we expressed *Rh1*^*G69D*^ under the control of *GMR-GAL4*. *Rh1*^*G69D*^ is a misfolded rhodopsin protein. The ‘Misfolded protein’ label refers to expression of *Rh1*^*G69D*^. **(A)** Knockdown of *SEL1L* reduces eye size in this ER stress model expressing *Rh1*^*G69D*^ in the eye. The Control fly is wildtype for *SEL1L*. (Eye size of Control 12.38 ± 0.93, *SEL1L* RNAi 10.93 ± 0.65, p = 0.0016). **(B)** In the ER stress model, with an *NGLY1* wildtype background, *SEL1L*^*S780P/+*^ (9.13 ± 0.49, p < 0.0001) and *SEL1L*^*Δ806-809/+*^ (9.29 ± 0.69, p < 0.0001) genotypes significantly increase eye size compared to the *SEL1L*^*+/+*^ genotype (8.36 ± 0.33). **(C)** Eye-specific knockdown of NGLY1 in the *Rh1*^*G69D*^ ER stress model results in no difference in eye size between the *SEL1L*^*+/+*^ and *SEL1L*^*S780P/+*^ genotypes. The *SEL1L*^*Δ806-809/+*^ genotype shows a significantly smaller eye size (p < 0.05). (Eye size: *SEL1L*^*+/+*^ 12.60 ± 0.50, *SEL1L*^*S780P/+*^ 12.34 ± 0.89, and *SEL1L*^*Δ806-809/+*^ 12.16 ± 0.65). **(D)** Heterozygous loss of *NGLY1* in the *Rh1*^*G69D*^ ER stress model results in no difference in eye size between the *SEL1L*^*+/+*^ and *SEL1L*^*S780P/+*^ genotypes. The *SEL1L*^*Δ806-809/+*^ genotype shows a significantly smaller eye size (p < 0.0001). (Eye size: *SEL1L*^*+/+*^ 10.58 ± 0.76, *SEL1L*^*S780P/+*^ 10.88 ± 0.61, and *SEL1L*^*Δ806-809/+*^ 8.67 ± 0.54).

We hypothesized that losing SEL1L would decrease ERAD and enhance the eye degeneration phenotype, leading to a smaller eye size. We expressed *SEL1L RNAi* in the eye discs to knockdown *SEL1L* in the ER stress model (and NGLY1 wildtype) and observed that the eyes were significantly smaller than in the flies without knockdown of *SEL1L* (p = 0.0016) (Fig 4A and S5 Data). The reduction in eye size observed in this model following *SEL1L* knockdown is expected, given SEL1L’s established role in ERAD function. We next crossed the different *SEL1L* variants onto this model (and NGLY1 wildtype) to investigate their effects on eye size. Because the ER stress model strain carries the common *SEL1L* wildtype allele, crossing our *SEL1L* variants onto this model provided the same *SEL1L* genotypes as previously described. We observed an increase in eye size of the *SEL1L*^*S780P/+*^ (p < 0.0001) and *SEL1L*^*Δ806-809/+*^ (p < 0.0001) genotypes compared to the SEL1L^*+/+*^ flies (Fig 4B and S5 Data), opposite of what we observed with the RNAi knockdown experiment. Given that previous studies show that improvement in ERAD function increases eye size, this result suggests that the *SEL1L*^*S780P*^ and *SEL1L*^*Δ806-809*^ alleles improve resistance to ER stress, likely through enhanced ERAD function, in an NGLY1 wildtype background.

We next tested whether ERAD is still improved by the *SEL1L*^*S780P*^ and *SEL1L*^*Δ806-809*^ alleles when NGLY1 activity is reduced. When we knockdown *NGLY1* in the eye of this ER stress model, eye size is no longer increased in the *SEL1L*^*S780P/+*^ and *SEL1L*^*Δ806-809/+*^ genotypes compared to the *SEL1L*^*+/+*^ (Fig 4C and S5 Data). This result suggests that the improvement in ERAD associated with the *SEL1L*^*S780P/+*^ and *SEL1L*^*Δ806-809/+*^ genotypes is dependent on NGLY1 activity. Heterozygous *NGLY1* null flies also showed no improvement in ERAD in the *SEL1L*^*S780P/+*^ and *SEL1L*^*Δ806-809/+*^ genotypes compared to the *SEL1L*^*+/+*^ genotype (Fig 4D and S5 Data). Interestingly, with the loss of NGLY1, the *SEL1L*^*Δ806-809/+*^ genotype had smaller eyes than the *SEL1L*^*+/+*^ genotype in both the *NGLY1* knockdown (p < 0.05) and heterozygous *NGLY1* null (p < 0.0001). Notably, our previous work has shown that *NGLY1* knockdown alone does not alter eye size in this ER stress model [7]. We conclude that the *SEL1L* variants only improve resistance to ER stress when wildtype NGLY1 activity is present, suggesting that NGLY1 is required to mediate ERAD-dependent or related protective mechanisms. In the absence or reduction of NGLY1, the *SEL1L* variants do not increase ERAD in this model. While these variants improved ER stress responses in the eye model, their effect on whole-organism survival under NGLY1 deficiency may reflect additional mechanisms beyond ERAD, such as altered handling of specific substrates like NRF1.

## Discussion

In this study, we sought to understand how natural SEL1L protein-coding variants identified in our *Drosophila* genetic screen influence NGLY1 deficiency lethality and other phenotypes. Our results demonstrate that these *SEL1L* variants increase survival in our NGLY1 deficiency models and protect against proteasome inhibition during both larval development and adulthood, although they do not mitigate larval size reductions induced by BTZ treatment. While these phenotypes are consistent with enhanced NRF1 activity, direct evidence for NRF1 involvement remains limited. Proteasome subunit gene expression measured from whole-body RNA suggests that the canonical NRF1-mediated bounce-back response is largely intact in heterozygous *NGLY1* null animals and is not influenced by the *SEL1L* variants. However, proteasome gene expression changes in specific tissues and at different developmental windows were not evaluated. Additionally, the increased sensitivity to low-dose bortezomib in our *SEL1L* variants (Fig 2B) could reflect altered responses to proteotoxic or ER stress beyond NRF1 signaling. Alternative pathways such as the unfolded protein response (UPR) [34], integrated stress response (ISR) [35], or autophagy [36] may contribute to the observed phenotypes. Given the central role of SEL1L in ERAD, it is also possible that enhanced clearance of specific ERAD substrates indirectly affects stress sensitivity or protein homeostasis [10]. While NRF1 remains a compelling candidate, our data do not exclude the contribution of other stress response mechanisms. Further studies will be needed to define the specific downstream effects of SEL1L variants on NGLY deficiency.

The results from our ER stress model indicate that the ability of these *SEL1L* variants to confer protection against ER stress, may involve enhanced ERAD function and requires NGLY1 activity. In our ER stress eye model, *SEL1L* variant effects on eye size were only observed in the presence of wildtype NGLY1. When *NGLY1* was reduced via knockdown or in a heterozygous *NGLY1* null, the improvement in eye size associated with *SEL1L*^*S780P*^ and *SEL1L*^*Δ806–809*^ alleles was lost, suggesting that ERAD enhancement by these variants requires NGLY1 activity. While ERAD is a separate pathway from NRF1 signaling, the pathways are inextricably linked [17–19,21]. We propose that our *SEL1L* variants may enhance ERAD efficiency, thereby increasing the amount of NRF1 that is retrotranslocated to the cytosol and available for activation. In heterozygous *NGLY1* null animals, where some NGLY1 activity remains, this may permit more efficient NRF1 activation and improved proteasome function. However, recent findings suggest that in the complete absence of NGLY1, NRF1 is not only inactive but may be diverted to a non-canonical ubiquitination pathway that exacerbates proteasome dysfunction [37,38]. Thus, the protective effects of the *SEL1L* variants we observe are likely contingent on residual NGLY1 activity and may not be generalizable to models of complete NGLY1 loss. While our data are consistent with enhanced NRF1 activation in the presence of the SEL1L variants, we did not directly measure NRF1 processing and nuclear localization. Therefore, the proposed involvement of NRF1 is a hypothesis based on prior literature, rather than a conclusion directly demonstrated in this study.

In addition to NRF1, recent work has shown that NGLY1 is required to deglycosylate misfolded BMP4, facilitating its removal from the ER and allowing properly folded BMP4 to dimerize and engage in canonical signaling [39]. BMP4 is required for proper developmental timing, so impairments in its signaling could affect larval size and growth [40]. Our previous studies have also demonstrated that proper folding and function of Ncc69/NKCC1 requires NGLY1 deglycosylation [9]. In these contexts, impaired clearance of misfolded NGLY1 clients could explain some of the phenotypes, and enhanced ERAD may restore proteostasis by clearing misfolded, misglycosylated proteins. Exploring these alternative mechanisms will be important for fully understanding the genetic interaction of *SEL1L* with *NGLY1*.

The SEL1L variants identified in our genetic screen are in the C-terminal cytoplasmic tail of the protein. While this region is not highly conserved across species, it is predicted, in all species, to be an intrinsically disordered domain [41]. Intrinsically disordered domains often mediate transient or multivalent protein–protein interactions and may contribute to dynamic complex formations [42–44]. Given SEL1L’s conserved role in ERAD, it is possible that these variants impact protein-protein interactions important for ERAD function and it will be important to determine whether analogous alterations in the cytoplasmic tail of mammalian SEL1L influence ER homeostasis or modulate phenotypes associated with NGLY1 deficiency. Functional studies in mammals could provide a deeper understanding of this poorly characterized domain of SEL1L and inform potential therapeutic targeting.

This study demonstrates that these naturally occurring SEL1L variants can enhance ERAD and mitigate specific phenotypes associated with NGLY1 deficiency. This raises the intriguing possibility that modulating SEL1L activity or ERAD efficiency could offer a therapeutic angle for this disease. However, further work is needed to determine whether these effects translate to mammalian systems and whether targeting ERAD components would improve proteostasis. It would also be interesting to test how modifications to mammalian SEL1L alter NRF1 processing or influence other stress response pathways. Ultimately, insights from cross-species modeling would help reveal whether modifying ERAD function can be leveraged to ameliorate disease phenotypes in NGLY1 deficiency.

Our findings implicate ERAD and proteasome function as potential therapeutic targets for NGLY1 deficiency patients. While there are still no therapeutic treatments for NGLY1 deficiency patients, there are two ongoing clinical trials, including an intracerebroventricular (ICV) *NGLY1* gene replacement therapy [45] and a GlcNAc supplementation trial to treat alacrima [46]. Further study is needed to investigate how enhancing ERAD affects the proteasome bounce-back response in NGLY1 deficiency patients who already suffer from proteasome dysfunction [37]. Most current therapeutics that target ERAD aim to decrease its function [47–49]; however, enhancing ERAD is possible in cells and animal models through increased expression of ERAD complex proteins [49,50]. In addition to SEL1L, our previous modifier screen has also identified other genes involved in ERAD or ER function, including *TMEM259*, *TMTC2*, and *ERMP1* [9]. Interestingly, for both *TMTC2* and *ERMP1*, our GWAS independently hit two separate fly orthologs of these genes [9]. ERAD components are strong candidates for modifier genes of NGLY1 deficiency patients and may be good drug targets for further development.

## Methods

### Fly stocks and maintenance

Stocks were maintained on standard agar-dextrose-yeast medium and standard Archon Scientific glucose fly food at 25˚C on a 12-h light/dark cycle. *SEL1L* CRISPR strains were created by WellGenetics, Inc (www.wellgenetics.com) and alleles were verified by Sanger sequencing (S3 Fig). The following stocks were obtained from the Bloomington *Drosophila* Stock Center (Bloomington, IN): Tubulin-GAL4 (BDSC #5138), UAS-pngl-RNAi (BDSC #54853), attp2 (BDSC #36303), and attp40 (BDSC #36304). The *Tubulin*-GAL80 strain was provided by Dr. Carl Thummel (University of Utah). The *SEL1L-RNAi* strain (v1161) was obtained from the Vienna *Drosophila* Resource Center [51]. The *NGLY1* null allele carries an early stop codon in the *NGLY1* gene, and was previously characterized and generously provided by Perlara, PBC [24,25]. The *NGLY1* null allele is homozygous lethal and the stock is maintained with the CyO balancer.

The “ER stress model” contains GMR-GAL4 and UAS-Rh1G69D on the second chromosome and has been previously described [31–33,52]. The endogenous *SEL1L* in this strain is homozygous for the *SEL1L*^*S780P*^ allele. We backcrossed the *SEL1L*^*S780*^ allele from our CRISPR generated *SEL1L*^*S780*^ strain into the ER stress model for 20 generations to create the desired homozygous *SEL1L*^*S780*^ genotype and verified genotype through sequencing.

### Proteasome sensitivity larval size assay

Proteasome sensitivity larval size assays were performed as previously described [25]. Standard *Drosophila* food was melted and cooled to 60˚C prior to the addition of DMSO or bortezomib. *NGLY1*^*+/-*^ females were pre-mated with males from a CRISPR *SEL1L* variant strain for 24 hours. Mated females were placed on food containing DMSO and allowed to lay eggs for approximately 8 hours. Adult flies were removed and after four days of development, 3^rd^ instar larvae were transferred to vials containing food with DMSO or bortezomib. After two days on bortezomib containing food, larvae were genotyped using the presence of GFP. The *CyO* balancer also carries a GFP marker. Larvae were confirmed to lack the balancer chromosome by checking that they were also GFP negative. Larvae were imaged at 2.5X magnification using a Leica EC3 camera. Larval size was quantified using ImageJ as previously described [24,25].

### Survival assays

NGLY1 knockdown (KD) eclosion survival: Virgin females from the CRISPR *SEL1L* variant strains were fed yeast overnight and then crossed with males from the donor strain UAS-NGLY1RNAi/Cyo,Tubulin-GAL80; Tubulin-GAL4/TM3,Sb. Progeny were collected and scored for the four balancer classes: CyO, Sb, double balanced, or no balancers, with the no balancer flies being the NGLY1 KD. This cross should produce the expected 1:1:1:1 ratio of the four genotypes. Given that there is always a very low level of lethality associated with each balancer, the largest balancer class was considered the closest to the expected number. We scored at least 200 flies per cross. Males and females were combined for a single count. To calculate the proportion of NGLY1 KD flies by generating a ratio of NGLY1 knockdown/largest balancer class as previously described [9]. For long-term survival, flies were collected, placed in a vial and flipped into fresh food every 2–3 days. Lifespan was measured as days post-eclosion. Vials were checked daily for dead flies and recorded.

### Eye imaging and quantification

Adult female flies aged 3–5 days were collected under CO2 anesthesia then frozen at -80°C for later imaging. Eyes were imaged at 3x magnification using the Leica EC3 Camera. Eye area was measured as previously described [31–33,53].

### Proteasome gene RT-qPCR

Changes in proteasome subunit gene expression were measured using RT-qPCR. Flies with appropriate *SEL1L* and *NGLY1* genotypes were crossed as previously described. 24 hours after eclosion, male flies were collected and placed on either 0.2% DMSO or 3μM BTZ for 12 hours or 24 hours. Immediately after drug treatments, RNA was extracted from 8-10 whole-body flies using a Direct-zol RNA Miniprep (Zymo Research R2061) using TRIzol Reagent (ThermoFisher Cat # 15596026) and including the DNAse step. RNA was converted to cDNA using a ProtoScript II First Strand cDNA Synthesis Kit (NEB Cat # E6560L). RT-qPCR was performed using a QuantStudio 3 96-well 0.2 ml block instrument and PowerUp SYBR Green Master Mix (ThermoFisher Cat #A25741). If available, we used primers from the FlyPrimerBank [54] located at http://www.flyrnai.org/flyprimerbank. Other primers were designed using Primer3Plus [55] located at www.primer3plus.com. All primer sequences listed in S4 Data. When comparing proteasome gene expression between the *NGLY1* wildtype and *NGLY1* null flies, gene expression was calculated relative to the *NGLY1* wildtype of each drug treatment and *SEL1L* genotype. In the comparison of proteasome gene expression changes with BTZ treatment, gene expression for all *SEL1L* genotypes was calculated relative to the DMSO treated *SEL1L*^*+/+*^ genotype.

## Supporting information

S1 Fig Wildtype *NGLY1* larvae are unaffected by proteasome inhibition. *NGLY1 WT* larvae treated with 1μM BTZ eclose as adults at similar rates regardless of *SEL1L* genotypes.
(TIF)

S2 Fig Proteasome gene expression changes in response to BTZ. (A) Gene expressions are shown relative to *NGLY1* wildtype for each drug treatment. No differences were observed in relative proteasome gene expressions between *NGLY1* WT and *NGLY1 + /-* flies. Top: *prosalpha6*; bottom: *prosbeta5*. (B) There is an increase in proteasome gene expression with BTZ treatment compared DMSO treatment. All values are shown relative to the “*SEL1L*^*+/+*^ DMSO” proteasome gene expression levels. Top: *prosalpha6*; bottom: *prosbeta5*. (**) p < 0.01, (*) p < 0.05.
(TIF)

S3 Fig Sequencing of CRISPR fly lines confirm appropriate *SEL1L* variants. Sanger sequencing of the three *SEL1L* CRISPR strains was used to verify the *SEL1L* alleles. (A) The top chromatograph is the *SEL1L* wildtype allele and the bottom is the *SEL1L*^*S780P*^ allele. The blue and red boxes show the base that is changed in the *SEL1L*^*S780P*^ variant allele. (B) The top chromatograph shows the unperturbed *SEL1L* gene. The box denotes the bases that are deleted, leading to the disruption of amino acids 806–809. The triangle on the bottom chromatograph shows where the deletion occurs in the *SEL1L*^*Δ806-809*^ allele.
(TIF)

S1 Data *NGLY1* ubiquitous knockdown fly counts and long-term survival. (XLSX)

S2 Data Larval size assay measurements. (XLSX)

S3 Data Fly counts for eclosion on BTZ and survival data. (XLSX)

S4 Data qPCR results and primers used. (XLSX)

S5 Data Fly eye measurements with ER stress model. (XLSX)
