# Supplementary figures and images for: Natural SEL1L variants rescue a model of NGLY1 deficiency and modify ERAD function and proteasome sensitivity
Source: PLoS Genet. 2025 Aug 7;21(8):e1011823. doi: 10.1371/journal.pgen.1011823 (PMC12342305; doi:10.1371/journal.pgen.1011823)

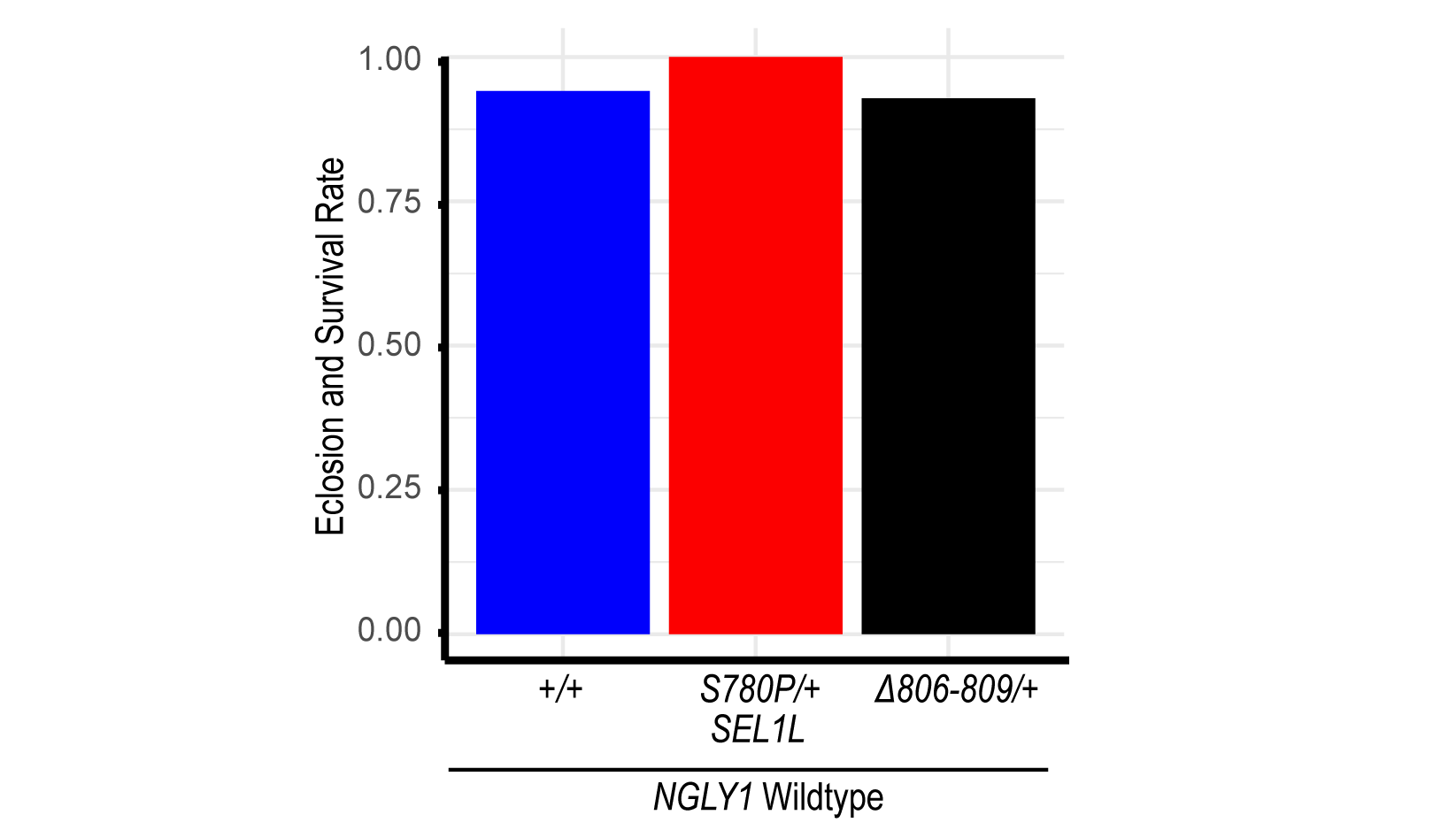

Supplement: S1 Fig — NGLY1 WT larvae treated with 1μM BTZ eclose as adults at similar rates regardless of SEL1L genotypes. (TIF) [file pgen.1011823.s001.tif]

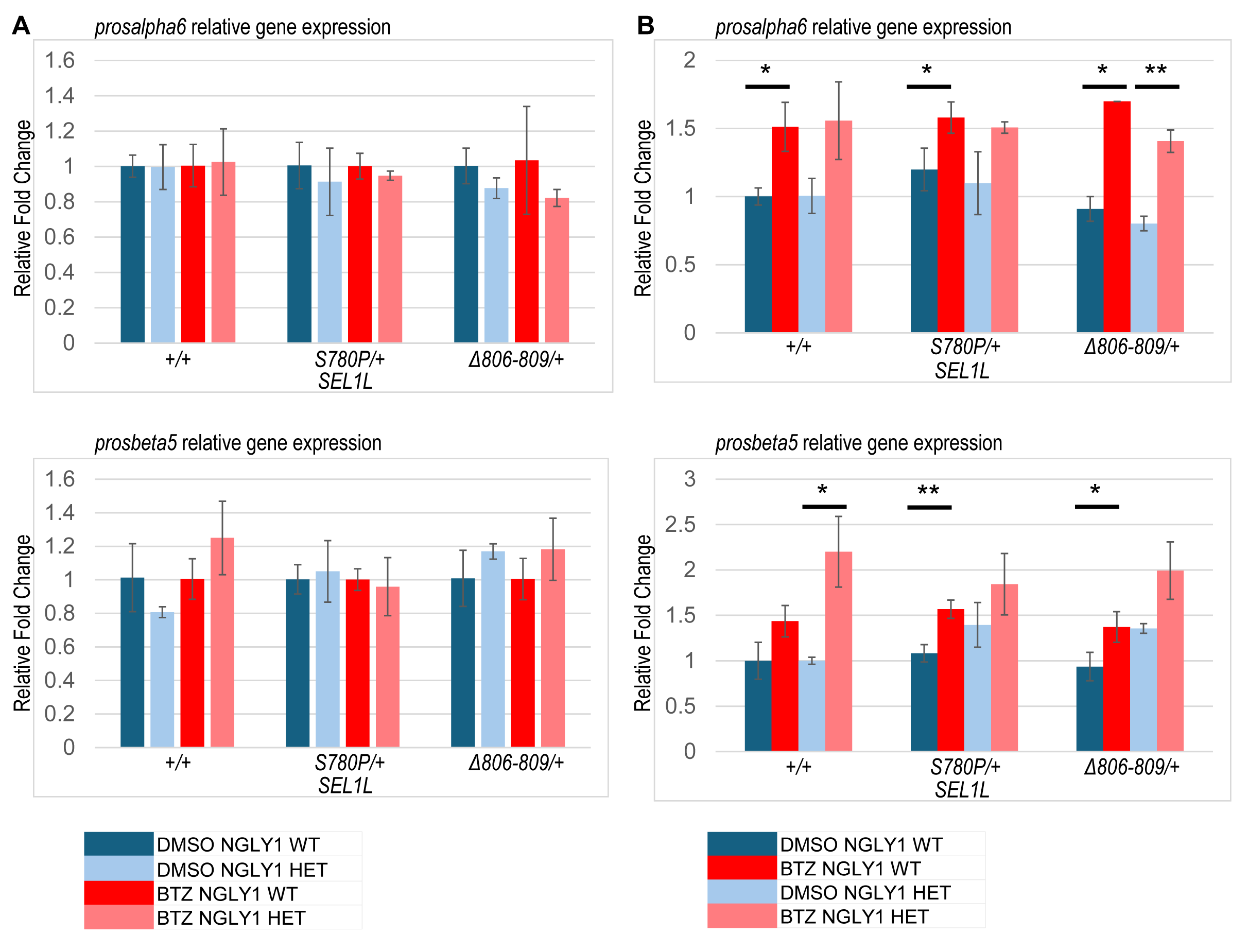

Supplement: S2 Fig — (A) Gene expressions are shown relative to NGLY1 wildtype for each drug treatment. No differences were observed in relative proteasome gene expressions between NGLY1 WT and NGLY1 + /- flies. Top: prosalpha6; bottom: prosbeta5. (B) There is an increase in proteasome gene expression with BTZ treatment compared DMSO treatment. All values are shown relative to the “SEL1L+/+ DMSO” proteasome gene expression levels. Top: prosalpha6; bottom: prosbeta5. (**) p < 0.01, (*) p < 0.05. (TIF) [file pgen.1011823.s002.tif]

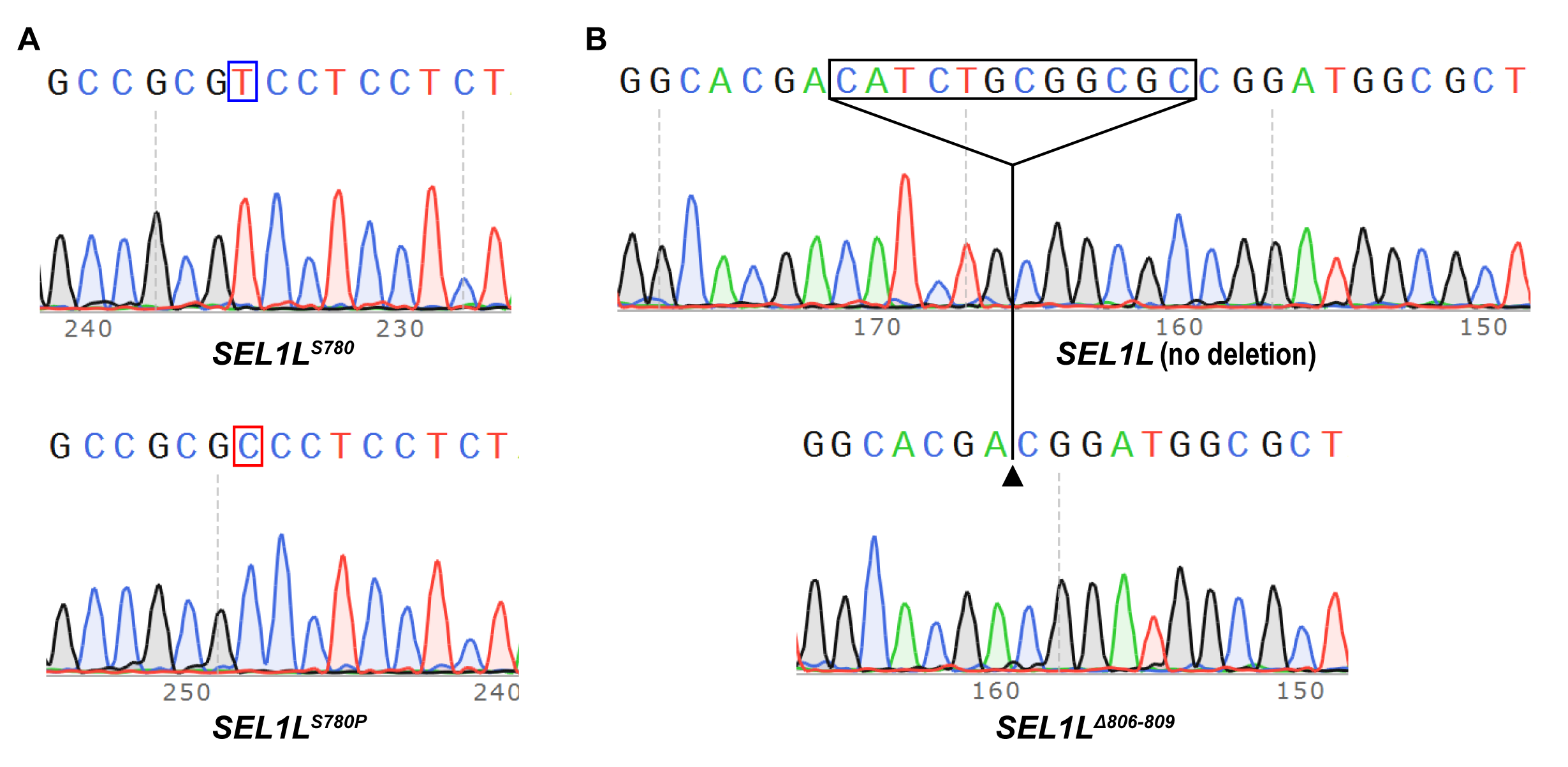

Supplement: S3 Fig — Sanger sequencing of the three SEL1L CRISPR strains was used to verify the SEL1L alleles. (A) The top chromatograph is the SEL1L wildtype allele and the bottom is the SEL1LS780P allele. The blue and red boxes show the base that is changed in the SEL1LS780P variant allele. (B) The top chromatograph shows the unperturbed SEL1L gene. The box denotes the bases that are deleted, leading to the disruption of amino acids 806–809. The triangle on the bottom chromatograph shows where the deletion occurs in the SEL1LΔ806-809 allele. (TIF) [file pgen.1011823.s003.tif]
